# Late cardiac events after allogeneic stem cell transplant: incidence, risk factors, and impact on overall survival

**DOI:** 10.1186/s40959-022-00150-1

**Published:** 2023-01-06

**Authors:** Christine Auberle, Daniel Lenihan, Feng Gao, Amanda Cashen

**Affiliations:** 1 Washington University School of Medicine, 660 South Euclid Avenue, Box 8056, St. Louis, MO 63110 USA; 2 International Cardio-Oncology Society, Tampa, FL USA; 3 St. Francis Healthcare, Cape Girardeau, MO USA

**Keywords:** Allogeneic stem cell transplant, Late cardiac events, Outcomes

## Abstract

**Background:**

There is limited data on the impact of cardiac disease on long term outcomes of allogeneic stem cell transplant (alloSCT). Our study aims to describe the incidence of late cardiac events after alloSCT, identify risk factors for developing a late cardiac event, and illustrate the impact of late cardiac events on overall survival.

**Methods:**

Patients who underwent alloSCT from 2007 to 2017 and survived more than 1 year after transplant (*N* = 804) were included. Gray’s sub-distribution methods, while accounting for death as a competing risk, were used to calculate the cumulative incidence of late cardiac events. Univariate regression models based on Gray’s sub-distribution were fitted to assess the potential predictive effects of baseline characteristics on the risk of developing any late cardiac events. Univariate Cox proportional hazard regression models were used to evaluate the association between late cardiac events and overall survival.

**Results:**

The cumulative incidence of a late cardiac event at 5 years after transplant was 22% (95% CI 19–25%). The most frequent cardiac event was a decline in LVEF to < 45% with a cumulative incidence of 9% (95% CI 7–11%). Patients were at significantly increased hazard of developing a late cardiac event if they had a history of congestive heart failure prior to alloSCT (HR 4.53, 95% CI 2.57–7.97, *p*-value < 0.001), a decline in LVEF to < 45% (HR 3.95, 95% CI 2.09–7.47, *p*-value < 0.001) or cerebral vascular accident (HR 3.13, 95% CI 1.38–7.06, p-value 0.004). Transplant characteristics such as primary disease, donor type, use of TBI, myeloablative conditioning regimen or tyrosine kinase inhibitor had no significant association with late cardiac events. Almost all cardiac events demonstrated a significantly increased risk of death. This hazard was the highest in patients who experienced an atrial arrhythmia (HR 10.6, 95% CI 7.7–14.6).

**Conclusion:**

Adverse cardiac events are relatively common late after alloSCT with identifiable risk factors such as medical comorbidities prior to transplant and are associated with a negative impact on overall survival.

## Background

Survival rates after allogeneic stem cell transplant (alloSCT) are improving as transplant methodology improves [1, 2], and patients who survive 2 years after transplant have greater than 80% probability of surviving to 10 years after transplant [3]. However, the relative mortality in patients receiving an alloSCT remains high even at 15 years after transplant [4]. Cardiac complications and their contribution to the relative increased mortality after hematopoietic stem cell transplant are of increasing importance.

To date, most studies examining cardiac complications in patients receiving hematopoietic stem cell transplant have been limited to the peri-transplant setting [5–7]. Few studies examine the cardiac complications occurring late after alloSCT, and those that have been published are limited by focus on a single type of cardiac event or by a small sample size [8–10]. In addition, there have been improvements in guidance for cardiac screening and monitoring in cancer patients. However, current guidelines recommending an electrocardiogram, echocardiogram, cardiac troponin and natriuretic peptides prior to therapy address all cancer types including a multitude of therapeutic exposures for both solid and hematologic malignancies [11]. Patients receiving alloSCT often undergo treatment prior to the transplant regimen and have a different set of risk factors associated with the transplant. Due to those differences and a gap in knowledge regarding the incidence of late cardiac events after alloSCT, further information is needed to develop specific recommendations regarding screening or monitoring in patients receiving alloSCT [12, 13].

Patients receiving alloSCT may be at higher risk for developing a late cardiac event than patients undergoing autologous stem cell transplant or non-transplant therapies. Systemic release of pro-inflammatory cytokines in patients after alloSCT may contribute to post-transplant complications [14]. Differences in therapeutic exposures compared to autologous stem cell transplant may also place alloSCT patients at higher risk of late cardiac events [15]. There is already evidence demonstrating alloSCT patients are at higher risk of diabetes, hypertension and dyslipidemia, well known risk factors for cardiac events [16–18].

Given the increasing number of alloSCT survivors and the higher probable risk for cardiac events, it is important to delineate risk factors in this patient population and to identify potential screening modalities for late cardiac events. Our study aims to describe the incidence of late cardiac events, their impact on overall survival and identify risk factors for developing a late cardiac event after alloSCT.

## Methods

Using the institutional transplant database, we identified 828 patients who underwent allogeneic stem cell transplant (alloSCT) between 2007 and 2017 at Siteman Cancer Center/Washington University in St. Louis, Missouri and survived more than 1 year after transplant. Twenty-four patients were excluded for inadequate follow up. If a patient received more than one alloSCT, data was collected for risk factors prior to and cardiac events following their first alloSCT. This study was conducted under the approval of the institutional review board of Washington University School of Medicine in St. Louis and informed consent was waived.

One abstractor collected all data with verification by a cardiologist specializing in Cardio-Oncology. Information on age at time of transplant, primary diagnosis, donor type, use of tyrosine kinase inhibitor, intensity of conditioning regimen, use of total body irradiation and date and cause of death was obtained from the transplant database at Siteman Cancer Center. Electronic medical records were used to abstract pre-alloSCT comorbidities, patient demographics and post-alloSCT late cardiac events.

Pre-alloSCT comorbidities that could impact the development of late cardiac events were recorded as documented at admission for alloSCT. Data was collected from diagnoses reported in past medical history, past surgical history and assessment and plan from history and physical notes as well as baseline electrocardiogram and echocardiogram prior to admission for alloSCT. Electrocardiogram and echocardiograms were interpreted by Cardiologists during admission. Pre-alloSCT risk factors included the following: body mass index, race, smoking status, hypertension, atrial arrhythmias, coronary artery disease, percutaneous coronary intervention (PCI), coronary artery bypass grafting (CABG), congestive heart failure (CHF), diabetes mellitus, and cerebral vascular accidents (CVA).

The primary endpoints in the study included the cumulative incidences of late cardiac events and overall survival (OS). Late cardiac events were defined as occurring more than 1 year after alloSCT up to December 31, 2019 and included: PCI, CABG, atrial or ventricular arrhythmias, a decline in left ventricular ejection fraction (LVEF) to < 45%, moderate to severe valvular disease, N-terminal pro B-type natriuretic peptide (NT-pro BNP) > 500 pg/mL, pericarditis, and moderate to severe pericardial effusion. Data was collected from diagnoses reported in office notes, inpatient consult notes and discharge summaries, cardiac catheterization reports, post-alloSCT echocardiograms and laboratory data. Overall survival (OS) was defined as the time from transplant to death due to any causes, and patients alive were censored at the date of last follow-up visit.

### Statistical analysis

Patient demographics and disease characteristics were summarized using counts and frequencies for categorical variables or means and standard deviations for continuous variables. The cumulative incidences of late cardiac events (both as a composite endpoint of any events and for each type of events individually) at 5-years post-transplant and their 95% confidence intervals were estimated using Gray’s sub-distribution methods, while treating deaths without cardiac events as competing risks. Univariate regression models based on Gray’s sub-distribution were fitted to assess the potential predictive effects of baseline characteristics on the risk of developing any late cardiac events [19]. A multivariate sub-distribution regression model with backward selection procedure was also performed to identify independent predictors, while adjusting all characteristics that were significant or marginally significant (*p* < 0.10) in the univariate analyses. Univariate Cox proportional hazard regression models were fitted to evaluate the association between late cardiac events (overall and for each type of events) and overall survival, while treating late cardiac events as time-dependent covariates. The effects of cardiac events on overall survival were summarized using hazard ratios (HR) and 95% confidence intervals. Kaplan-Meier curves were also created to graphically present the effect of cardiac events on overall survival. Specifically, for patients who developed cardiac events, the time-0 of follow-up was re-set as the onset of cardiac events. For patients without cardiac events, the time-0 of follow-up was set at 30 months post alloSCT (i.e., the median onset time of cardiac events), while those without cardiac events who died before 30-month were excluded from the Kaplan-Meier curve [20]. All tests were two-sided and significance was set at a *p*-value of 0.05. All the analyses were performed using SAS 9.4 software (SAS Institutes, Cary, NC, USA).

## Results

### Patient characteristics

Of patients receiving an alloSCT between 2007 to 2017, 804 patients survived more than 1 year after transplant and were included in the analysis. Patient characteristics are shown in Table 1. The average age at time of transplant was 49.7 years old with the majority of patients between 40 and 59 years of age. Of patients surviving more than 1 year after alloSCT, 466 (58%) were still living at time of data abstraction and 428 (92%) of those had an available medical record within 12 months of the censor date. The average body mass index was 28.8 kg/m^2^ and 26% of patients were smokers at time of transplant. The majority of patients (93%) identified as non-Hispanic white. The most common pre-alloSCT medical comorbidities were hypertension in 227 (28%), diabetes in 80 (10%), and coronary artery disease in 46 (6%) patients.

**Table 1:** Characteristics

	***N*** = 804
**Sex**	
Male	472 (59%)
Female	332 (41%)
**Age**	
Age 18–39	182 (23%)
Age 40–59	406 (51%)
Age >/= 60	216 (27%)
Mean age	49.7 years
Mean body mass index	28.8 kg/m^2^
Current smoker	207 (26%)
**Race/Ethnicity**	
Non-Hispanic White	749 (93%)
Black	38 (5%)
Asian	13 (2%)
Other/unknown	4 (1%)
**Primary Diagnosis**	
Acute myeloid leukemia	401 (50%)
Myelodysplastic syndrome	132 (16%)
Acute lymphoblastic leukemia	91 (11%)
Lymphoma	72 (9%)
Chronic lymphocytic leukemia	37 (5%)
Chronic myeloid leukemia	37 (5%)
Aplastic Anemia	23 (3%)
Myeloma	7 (1%)
Other	4 (1%)
**Transplant Characteristics**	
Myeloablative conditioning regimen	598 (74%)
Total body irradiation	278 (35%)
Tyrosine Kinase inhibitor prior to transplant	62 (8%)
**Donor Type**	
Matched unrelated	442 (55%)
HLA identical sibling	275 (34%)
Haploidentical	75 (9%)
Mismatched unrelated	10 (1%)
**Pre-transplant Medical Comorbidity**	
Hypertension	227 (28%)
Diabetes	80 (10%)
Coronary artery disease	46 (6%)
Atrial arrhythmia	41 (5%)
PCI or CABG	36 (5%)
LVEF < 45%	16 (3%)
Congestive heart failure	20 (3%)
Valvular disease	14 (2%)
Cerebral vascular accident	9 (1%)

Bolded text represents subheading

*CABG* coronary artery bypass grafting, *HLA* human leukocyte antigen, *LVEF* left ventricular ejection fraction, *PCI* percutaneous coronary intervention

Half of patients (50%) had acute myeloid leukemia as their primary diagnosis for transplant with myelodysplastic syndrome (16%) and acute lymphoblastic leukemia (11%) being the next most common primary diseases. 74% of patients received a myeloablative conditioning regimen, 35% received total body irradiation (TBI) and 8% of patients received a tyrosine kinase inhibitor. Matched unrelated donors were used in 55% of transplants (Table 1).

### Late cardiac events

**Fig. 1:**
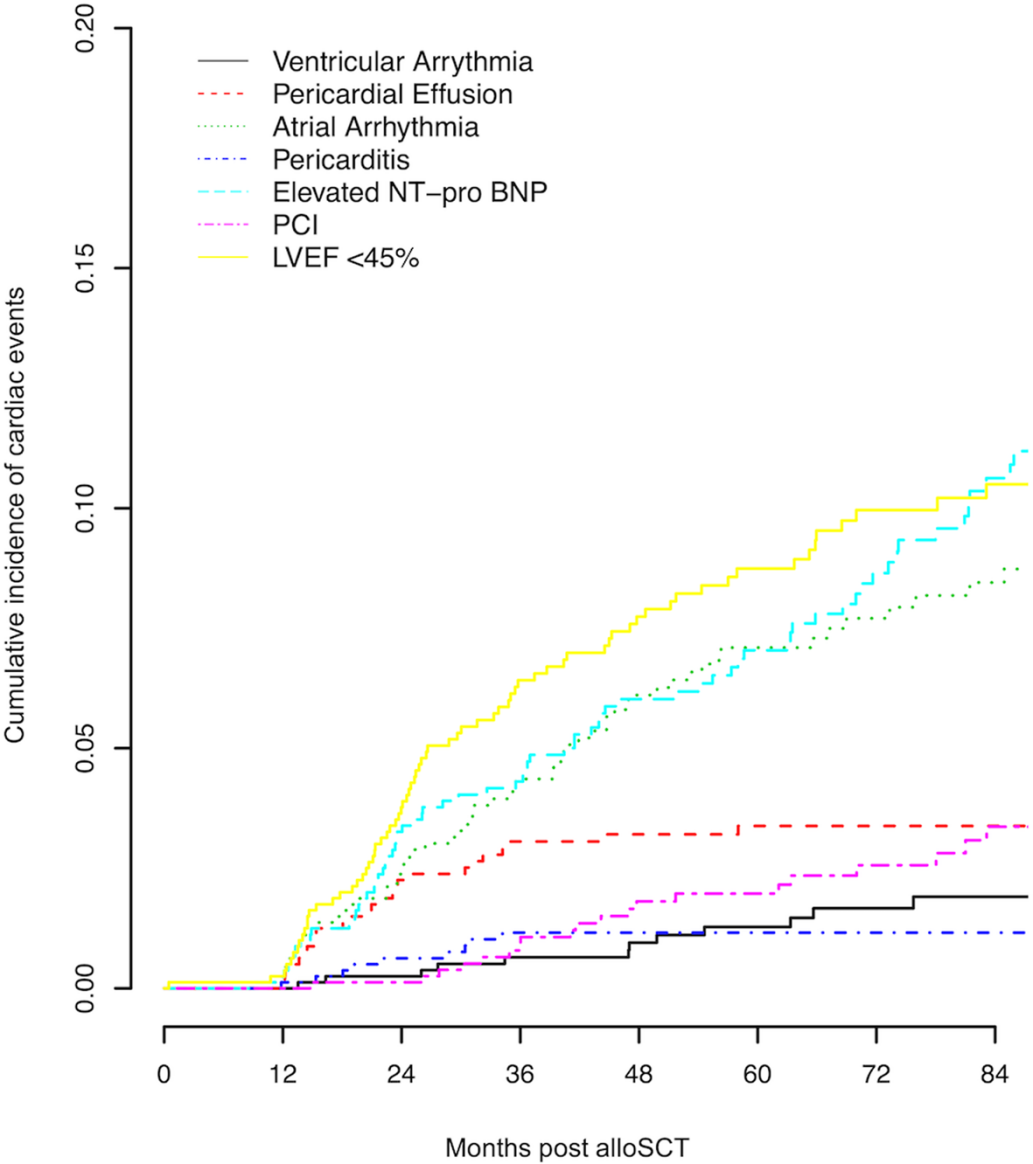
Cumulative Incidence of Cardiac Events After AlloSCT. Cumulative incidence of each cardiac event from 1 to 7 years after alloSCT. LVEF = left ventricular ejection fraction; PCI = percutaneous coronary intervention

At 5 years after transplant the cumulative incidence of any cardiac event was 22% (95% CI 19–25%) (Table 2). The most frequent cardiac event was a decline in LVEF to < 45% with a cumulative incidence of 9% (95% CI 7–11%). Figure 1 demonstrates the cumulative incidence of adverse cardiac events one to 7 years after alloSCT (Fig. 1).

**Table 2:** Cumulative incidence of late cardiac events at 5 years after alloSCT

Cardiac Events	Incidence	95% CI
Any cardiac event	22%	19–25%
LVEF < 45%	9%	7–11%
Atrial arrhythmia	7%	5–9%
Elevated NT-pro BNP	7%	5–9%
Pericardial effusion	3%	2–5%
PCI	2%	1–3%
Ventricular arrhythmia	1%	0.6–2%
Pericarditis	1%	0.6–2%

*AlloSCT* allogeneic stem cell transplant, *LVEF* left ventricular ejection fraction, *NT-pro BNP* N-terminal pro B-type natriuretic peptide, *PCI* percutaneous coronary intervention

The patient characteristics that are significantly associated with an increased hazard of developing any late cardiac events are male sex (HR 1.43, 95% CI 1.06–1.92, *p*-value 0.017), increasing age (HR 1.03, 95% CI 1.02–1.05, *p*-value < 0.001) and BMI (HR 1.02, 95% CI 1.00–1.04, p-value 0.026). Several medical comorbidities are strongly associated with late cardiac events, including CHF (HR 4.53, 95% CI 2.57–7.97, *p*-value < 0.001), LVEF < 45% (HR 3.95, 95% CI 2.09–7.47, *p*-value < 0.001) and history of CVA (HR 3.13, 95% CI 1.38–7.06, *p*-value 0.004) (Table 3).

**Table 3:** Association between patient characteristics and developing late cardiac events

Characteristic	Hazard Ratio (95% CI)	Log-rank ***P***-value
Male	1.43 (1.06–1.92)	0.017^*^
Age (per 1-year increase)	1.03 (1.02–1.05)	<.001^*^
Body mass index (per 1-kg/m^2^ increase)	1.02 (1.00–1.04)	0.026^*^
Smoker	0.87 (0.62–1.21)	0.401
History of congestive heart failure	4.53 (2.57–7.97)	< 0.001^*^
History of LVEF < 45%	3.95 (2.09–7.47)	< 0.001^*^
History of cerebral vascular accident	3.13 (1.38–7.06)	0.004^*^
History of coronary artery disease	2.54 (1.61–4.00)	< 0.001^*^
History of PCI or CABG	2.28 (1.35–3.87)	0.002^*^
History of diabetes	1.70 (1.12–2.57)	0.011^*^
History of hypertension	1.65 (1.23–2.20)	< 0.001^*^
History of arrhythmia	1.45 (0.79–2.67)	0.229
History of valvular disease	1.42 (0.58–3.44)	0.441

*CABG* coronary artery bypass grafting, *LVEF* left ventricular ejection fraction, *PCI* percutaneous coronary intervention

^*^statistically significant

In multivariate analysis, age (HR 1.03, 95% CI 1.02–1.04, *p*-value < 0.001) and BMI (1.02, 95% CI 1.00–1.05, p-value 0.026), as well as three comorbidities, including LVEF < 45% (HR 2.71, 95% CI 1.23–5.97, p-value 0.013), CHF (HR 2.52, 95% CI 1.25–5.06, p-value 0.009) and coronary artery disease (HR 1.64, 95% CI 1.02–2.64, p-value 0.040), remained significant. Transplant characteristics such as primary disease, donor type, and use of TBI, myeloablative conditioning regimen or tyrosine kinase inhibitor had no significant association with late cardiac events.

### Overall survival

**Fig. 2:**
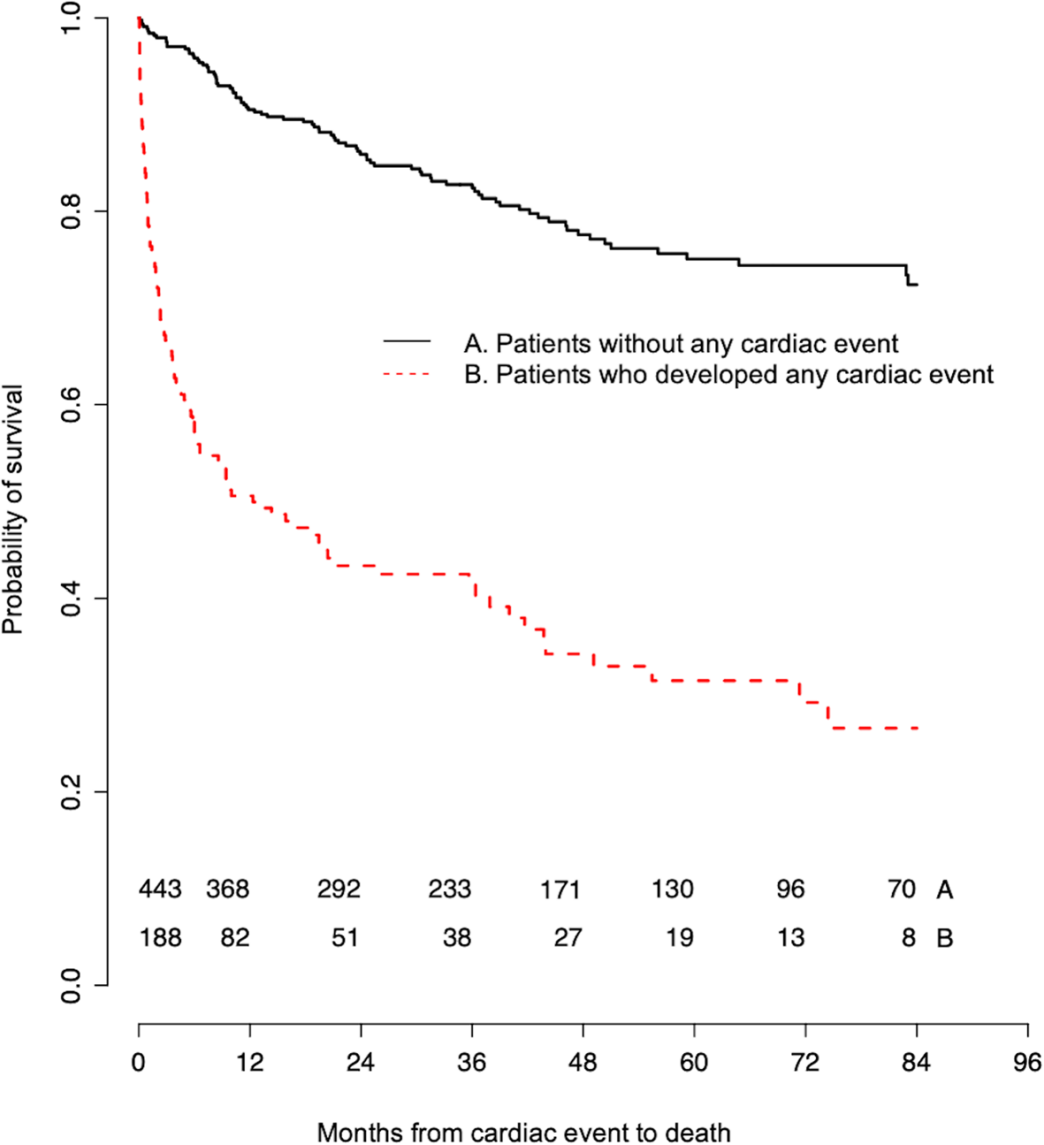
Effects of late cardiac events on overall survival. Kaplan-Meier curves demonstrating the effect of any cardiac event on overall survival

Our analysis found late cardiac events have a significant negative impact on overall survival (Table 4). Almost all cardiac events, with the exception of PCI, demonstrated a significantly increased risk of death. This hazard was the highest in patients who experienced an atrial arrhythmia (HR 10.6, 95% CI 7.7–14.6). A graphical presentation for the impact of any late cardiac events on overall survival is presented in Fig. 2, illustrating the time from cardiac events until death (Fig. 2).

**Table 4:** Effect of late cardiac events on risk of death

Cardiac event	Hazard Ratio (HR)	95% CI
Atrial arrhythmia	10.6^a^	7.7–14.6
Pericarditis	7.5^a^	3.9–14.7
LVEF < 45%	5.8^a^	4.3–7.9
Elevated NT pro-BNP	5.2^a^	3.7–7.3
Pericardial effusion	5.2^a^	3.3–8.3
PCI	1.6	0.7–3.7

*LVEF* left ventricular ejection fraction, *NT-pro BNP* N-terminal pro B-type natriuretic peptide, *PCI* percutaneous coronary intervention

^a^statistically significant

## Discussion

Cardiovascular complications have been described as the most common late complication after allogeneic transplant [21], and patients who have received an alloSCT have 2.3 fold higher risk of premature cardiovascular death [4]. We examined the incidence and risk factors of late cardiac events and their impact on overall survival in patients who received an alloSCT. To our knowledge no other studies have examined multiple cardiac endpoints occurring in alloSCT survivors. Other studies have been limited in their smaller sample sizes or inability to follow up with patients in the years after the transplant. Given the retrospective, single-center nature of this study, we were able to analyze long-term follow up data and abstract data for multiple cardiac events for one of the largest populations of alloSCT survivors ever examined.

We found that adverse cardiac events more than 1 year after alloSCT are relatively common, with almost a quarter of patients experiencing a late cardiac event. The events with the highest cumulative incidence were markers for heart failure including a reduced LVEF and elevated NT-pro BNP. In patients with multiple comorbidities, a markedly elevated BNP may be present in a patient with heart failure even without significant symptoms of volume overload or left ventricular dysfunction and is associated with a higher risk of mortality [22]. Previous reports describe a timeline for developing late CHF after autologous and allogeneic transplants with the majority of heart failure diagnoses occurring in the first 4 years after transplant [23]. In our patient population, we found a similar logarithmic trend to the cumulative incidence of reduced LVEF. The cumulative incidence of an elevated NT-pro BNP presented a more linear pattern, suggesting the risk for developing an elevated NT-pro BNP continues late after transplant.

The risk of developing cardiac arrhythmias after transplant has been uncertain [24]. A study of 1491 patients in the state of Washington reported approximately a 5% cumulative incidence of rhythm disorders at 5 years after transplant and a higher risk of developing a rhythm disorder compared to the general population [25]. The study did not delineate between atrial and ventricular arrhythmias and were documented only if the patient required hospitalization, suggesting a possible underestimation of the burden of arrhythmias. The cumulative incidence of atrial arrythmias in our population was slightly higher at 7% 5 years after transplant. The incidence increased in a linear trajectory in the following years after transplant, which is logical given advancing age is one of the largest risk factors for the development of atrial arrhythmias [26]. The cumulative incidence of obstructive coronary artery disease requiring intervention such as PCI was also consistent with other studies. A multi-center European study reported approximately 1% cumulative incidence of arterial events at 5 years after alloSCT [27]. Another study, reports similar values of cumulative incidence of an arterial event of 1.5% at 5 years [28].

While characteristics such as male sex, increasing age and BMI demonstrate a higher risk of developing a late cardiac event, the highest risk is found in patients with medical comorbidities. Particularly in patients with a history of CHF or findings on echocardiogram of reduced left ventricular function as well as those with arterial disease resulting in cerebral vascular accidents, coronary artery disease, PCI, and CABG. While patients who have not undergone alloSCT with these risk factors are also at risk for further events, given the higher risk of death from cardiac events in the alloSCT population, pre-transplant risk factors should be used to determine monitoring for cardiac events in the post-transplant setting. Another study emphasizes the importance of pre-transplant risk factors by describing post-transplant cardiovascular risk factors as approaching a similar risk as pre-transplant factors in developing ischemic heart disease after having been sustained for greater than 1 year after transplant [29]. Patients with risk factors prior to alloSCT for cardiac events may benefit from closer monitoring in the post-transplant setting.

Intensity of conditioning regimen and use of radiation during transplant have been cited as possible sources of cardiotoxicity resulting in late cardiac events [30]. However, transplant characteristics such as primary disease, donor type, and use of TBI, myeloablative conditioning regimen or tyrosine kinase inhibitor did not significantly increase the risk of developing late cardiac events in our patient population. A patient’s characteristics and medical comorbidities have a greater impact in the development of late cardiac events than therapy related to the alloSCT. This finding has been supported in other studies examining individual cardiac events such as arterial disease or CHF [23, 27, 29].

As with any retrospective review, there were some limitations. As our data was collected from medical records, we were limited by the information available within those records and lack of consistency in documentation of past medical conditions or among interpretation of echocardiograms. This may result in underreporting of medical comorbidities prior to transplant or cardiac events following transplant. Given the span of our retrospective review and the advancements in biomarkers, such as NT-pro BNP, there may also have been under-utilization of these tests in patients from our analysis transplanted earlier resulting in an underestimation of the burden of cardiac events. Finally, our study was performed at a single institution and there may be geographic attributes to our population that do not apply to others.

## Conclusion

In summary, cardiac events are relatively common late after alloSCT, and have a high risk of decreasing overall survival in alloSCT patients. Those at highest risk for experiencing an adverse cardiac event are those with medical comorbidities prior to transplant. Transplant characteristics do not have a significant association with the development of late cardiac events. Screening methodologies in alloSCT survivors, such as echocardiograms and cardiac biomarkers, and early interventions may be important in reducing the risk of cardiac events on overall survival and improving patient outcomes and worthy of future exploration.

## Data Availability

The datasets generated and/or analysed during the current study are available from the corresponding author on reasonable request.

## Abbreviations

alloSCT: Allogeneic stem cell transplant
CABG: Coronary artery bypass grafting
CHF: Congestive heart failure
CVA: Cerebral vascular accident
DM: Diabetes mellitus
HTN: Hypertension
NT-pro BNP: N-terminal pro B-type natriuretic peptide
PCI: Percutaneous coronary intervention
TBI: Total body irradiation
TKI: Tyrosine kinase inhibitor
